# Insecticides for Suppression of *Nylanderia fulva*

**DOI:** 10.3390/insects8030093

**Published:** 2017-08-31

**Authors:** Dawn Calibeo, Faith Oi, David Oi, Catharine Mannion

**Affiliations:** 1 Gowan Company, LLC; Yuma, AZ 85365, USA; dcalibeo@gowanco.com; 2 Department of Entomology and Nematology, University of Florida, Gainesville, FL 32611, USA; 3 Center for Medical, Agricultural and Veterinary Entomology, United States Department of Agriculture Agricultural Research Service (USDA ARS), Gainesville, FL 32608, USA; David.Oi@ARS.USDA.GOV; 4 Tropical Research & Education Center, Department of Entomology and Nematology, University of Florida, Homestead, FL 33031, USA; cmannion@ufl.edu

**Keywords:** *Nylanderia fulva*, tawny crazy ant, suppression, control

## Abstract

*Nylanderia fulva* (Mayr) is an invasive ant that is a serious pest in the southern United States. Pest control operators and homeowners are challenged to manage pest populations below acceptable thresholds. Contact and bait insecticides are key components of an Integrated Pest Management (IPM) strategy, however, little is known about their efficacy. In repellency and efficacy bioassays, *N. fulva* were not completely repelled by any insecticide tested, although fewer ants crossed a surface treated with Temprid^®^. Few insecticides provided rapid control. Termidor^®^ and Temprid^®^ were the best performing with mean mortality of 100% in 13.4 and 19.0 days, respectively. In no-choice bait acceptance studies, it was shown that *N. fulva* generally had greater acceptance of carbohydrate-based ant baits (Advion^®^, InTice^TM^ (gel), and InTice^TM^ (granular)). However, mortality was low for the InTice^TM^ baits in a 7-day bioassay. Maxforce^®^ Ant Killer Bait Gel and Advance^®^ 375A in the spring and Maxforce^®^ Complete in the summer and fall required the fewest days to reach 100% mortality. Bait active ingredients that resulted in the highest mortality were hydramethylnon and fipronil. These data on the efficacy of commercially available contact and bait insecticides provide valuable information to manage this invasive pest.

## 1. Introduction

*Nylanderia fulva* (Mayr) (Hymenoptera: Formicidae) is an invasive ant that is a serious pest in the southern United States. Already established as an urban and ecological pest, this ant has the potential to be an agricultural and industrial pest as well. Infestations of *N. fulva* may be quite large and in some cases ants are dispersed over entire neighborhoods [1]. One reported infestation of *N. fulva* in Colombia was >10 ha [2]. LeBrun et al. [3] reported that introduced *N. fulva* may reach numerical abundance greater than 2 orders of magnitude over all other ants. In the southern United States, when occurring at high densities, *N. fulva* displaces most of the larger ant species including *Solenopsis invicta* Buren. *Nylanderia fulva* also significantly lowers species richness and abundance of non-ant arthropods in the invaded area [3]. Over a period of years, *N. fulva* populations decline, even without pest control intervention [2,4], but the reasons for population reductions have not yet been elucidated. In the meanwhile, there is a great need for information on management of *N. fulva*.

To date there have been few published studies on *N. fulva* control. Spraying and baiting are two application methods commonly used to control ants. Barrier treatments are applications of liquid or granular insecticides around the exterior of structures to prevent or restrict ants from entering the structure [5,6,7]. Crack and crevice treatments are indoor applications of liquid insecticides to areas that provide harborages or nesting sites for ants. These structural voids also may be treated with a desiccant dust or a foam formulation of a liquid insecticide.

Both liquid sprays and baits are important to integrated pest management (IPM) strategies; however, efficacy data are often lacking. Applications of some insecticides to the exterior perimeter of a structure are effective at killing thousands of *N. fulva* [1]. However, if the cadavers are not removed, live ants simply use them as a bridge over the treated area. The immense numbers of ants in a typical infestation, homeowner actions to remove dead ants such as washing down patios and sidewalks, intense solar radiation, high humidity, and seasonal daily rainfall present challenges to the residual efficacy of insecticides applied for *N. fulva* control.

Data on *N. fulva* bait effectiveness also are scant. The use of insecticidal baits instead of broadcast applications of contact insecticides to control invasive species may have a positive impact on the abundance and diversity of native ants [8]. Another advantage is that baits do not have to be collected by all foraging ants to provide effective control. Bait toxicants are transferred via trophallaxis to nestmates within the colony [9].

Drees et al. [10] investigated *N. fulva* acceptance of some commercially available granular baits. Advance^®^ Carpenter Ant Bait (abamectin, BASF, Research Triangle Park, NC, USA) and Maxforce^®^ Complete Ant Bait (hydramethylnon, BASF, Research Triangle Park, NC, USA) were more accepted by *N. fulva* than Amdro^®^ Ant Block (hydramethylnon, Central Garden and Pet, Atlanta, GA, USA), ProBait^®^ (hydramethylnon, Zoecon, Shaumburg, IL, USA), Extinguish^®^ Plus (hydramethylnon and methoprene, Wellmark International, Schaumburg, IL, USA), and Esteem^®^ (pyriproxyfen, Valent USA Corporation, Walnut Creek, CA, USA). Advance^®^ Carpenter Ant Bait was tested against *N. fulva* in a large field trial in Texas, but it did not adequately suppress ant populations [11]. Also in Texas, one field application of Esteem^®^ Ant Bait was not effective against *N. fulva*, as ants from nearby untreated areas re-infested the treated area within 14 days [12].

The objectives of this study were to identify potential products for inclusion in an IPM strategy for suppression of *N. fulva* by (1) evaluating the repellency and efficacy of commercially available professional contact insecticide products used as sprays and (2) to evaluate the acceptance and efficacy of commercially available ant baits in laboratory assays.

## 2. Materials and Methods

### 2.1. Ant Colonies

**Figure 1:**
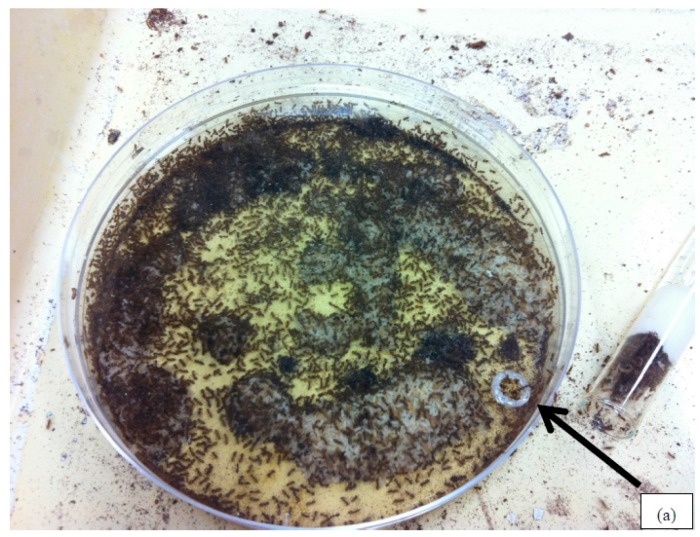
Artificial nest for laboratory maintained *Nylanderia fulva* colony fragments. The artificial nest consists of a plastic petri dish (100 × 15 mm) filled three-quarters with dental plaster. A hole (a) melted into the petri dish cover allowed ants to access to the interior of the nest.

*Nylanderia fulva* were collected from one of two urban sites in Gainesville, Florida, each with an infestation that had been established for at least 2 years prior to the study. Ant collection was achieved by locating a colony fragment in leaf litter and quickly scooping the leaf litter and a thin layer of the underlying soil into a plastic container. The interior walls of the plastic container were coated with Insect-a-Slip^®^ (BioQuip Products, Inc., Rancho Dominguez, CA, USA), a slippery coating that prevents ants from escaping the container. Care was taken to collect workers, brood, and queens quickly, as *N. fulva* will relocate immediately upon being disturbed. Containers with collected ants were transported to the laboratory where the ants were provided with an artificial nest constructed from a round polystyrene petri dish (100 × 15 mm) filled three-quarters with dental plaster and with a hole (3 mm) melted into the cover to allow entry (Figure 1). As the leaf litter dried, the ants relocated into the artificial nest and the litter was gradually removed, allowing for easy observation and harvesting of ants from the colony fragments. The ants were fed live termites and/or slices of canned sausage (Armour^®^ brand, Pinnacle Foods Group, LLC, Cherry Hill, NJ, USA) every other day. Water and 10% sucrose solution were provided ad libitum via test tubes stoppered with cotton.

### 2.2. Liquid Insecticide Repellency and Efficacy Bioassays

**Figure 2:**
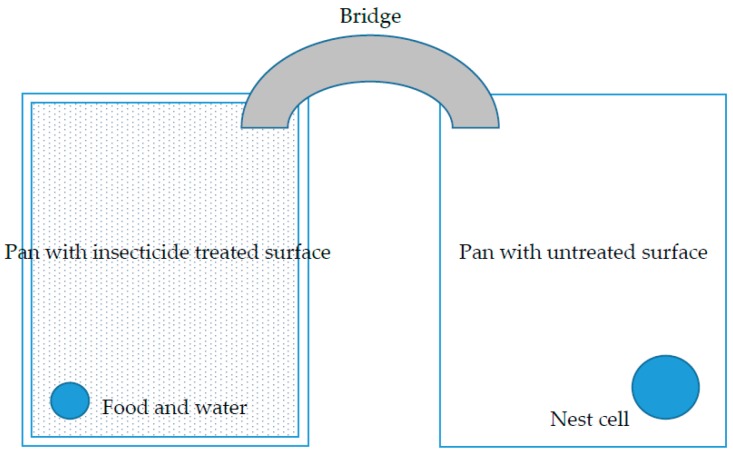
Experimental set-up of laboratory bioassay evaluating repellency and efficacy of commercially available insecticides against *Nylanderia fulva*. The interior dimensions of each arena were 31 × 21 × 5 cm. Insecticides were applied to a painted wood panel that was then attached to the bottom of one pan (shaded area). An artificial nest with 2000 workers and 15 queens (no brood) was placed into the untreated pan. Worker ants were required to cross the length of the insecticide treated arena for food and water.

Test arenas (Figure 2) were designed to simulate ants crossing an insecticide treated barrier. Arenas were prepared by coating the inside walls of rectangular aluminum pans (31 × 21 × 5 cm) with Insect-a-Slip. Panels of plywood (0.6 cm thick) were cut to fit the inside bottom of each tray. To simulate the exterior surface of a structure, the panels were painted with white latex paint (Behr Premium Plus, Masco Corporation; Taylor, MI, USA) and allowed to dry for 24 h.

Nine commercially available insecticides (Table 1) were diluted in water according to each product’s manufacturer label directions at the highest concentration allowed for exterior application for ants and tested in two groups. Termidor^®^, Temprid^®^, Suspend^®^ and an untreated control were tested first, then Arilon^®^, Ortho^®^, Optigard^®^, Phantom^®^ SC, Phantom^®^ aerosol, and Talstar^®^ with an untreated control. The first group were contact insecticides commonly used by pest control companies at the time of testing. The second group of insecticides were ones that emerged as additional products being used by the pest control industry. All treatments were replicated five times. The insecticide dilutions were agitated thoroughly and decanted into disposable plastic spray bottles.

Label directions for application volume varied by product or were absent. Therefore, the insecticide dilutions or deionized water (control) were applied to the plywood panels until wet, simulating an outdoor perimeter application that might be performed by a pest control operator. The panels were allowed to dry for 24 h. Once dry, the panels were attached to the inside bottom of the aluminum pans with foam tape. To prevent ants from going under the panels, the edges were sealed to the aluminum pans with caulking. Caulking was allowed to cure for 24 h.

Large plastic trays were prepared as secondary containment vessels by coating the inside walls with Insect-a-Slip. One aluminum pan with an insecticide treated panel (treated surface) was placed inside the plastic tray along with one aluminum pan with Insect-a-Slip coated interior walls only (untreated surface).

An index card was placed on end into a container with field collected *N. fulva*. Ants were allowed to climb onto the card, counted, and gently tapped into a new container. Approximately 2000 *N. fulva* workers and 15 queens were collected from the same colony and placed into each untreated surface pan. Brood was not included in the bioassay. The ants were provisioned with a nest cell as described above and a test tube filled with deionized water stoppered with cotton. The ants were starved for 24 h before the test began. Additionally, a black plastic container (15.2 × 7.6 × 1.6 cm) was notched on one end and placed upside down over the nest cell to provide additional harborage and to maintain a humid microclimate. After 24 h, the water tube was removed from the untreated surface pan and placed into the treated surface pan along with live termites and a test tube containing 10% sucrose solution stoppered with cotton. A bridge constructed of wire fabric wrapped with aluminum tape was used to connect the two pans. The food and water were placed so that foraging ants had to cross the entire distance of the treated panel to obtain food or water (Figure 2).

To assess the relative repellency of the insecticides, the number of *N. fulva* crossing a fixed point on the treated surface in the direction of the food was recorded. Ants were counted for 60 s every 30 min for the first 2 h and summed to arrive at a foraging count. Each of the two sets of insecticides was analyzed separately using a completely randomized design. Repellency data met the assumptions of normality, homoscedasticity, and independence as evaluated by Shapiro Wilk, Levene’s, and Durbin-Watson tests, respectively. For each experiment, foraging counts among the insecticides were compared with a one-way analysis of variance (ANOVA) and Tukey’s HSD test (α = 0.05) [13].

To evaluate the efficacy of each insecticide, the number of dead ants for each colony fragment was recorded daily for 30 days. For each experiment, cumulative percent mortality was calculated at 10 days and at 17 and 28 days for experiments 1 and 2, respectively. Cumulative percent mortality was calculated by dividing the cumulative number of dead ants by 2000, the approximate number of ants placed into the untreated pan at the beginning of the study. The data for each experiment did not meet the assumptions of ANOVA, thus rank-scores were used. Ranks, “3” = 90–100% mortality, “2” = 50–89% mortality, and “1” = 0–49% mortality, were selected to indicate high, medium, and low mortality, respectively. A period of 10 days was selected to allow some of the slower acting products time to impact the ants, while 17 days or 28 days was selected because (1) this is the point where control mortality approached 20% and (2) in the case of 28 days, it is close to the common pest control service interval (i.e., monthly service). Rank-scores were compared among treatments with a one-way ANOVA and Tukey’s HSD test (α = 0.05) [13,14] for each experiment when appropriate.

### 2.3. Bait Acceptance and Efficacy Assays

For the bait acceptance and efficacy tests, worker ants from laboratory colonies were placed in groups of twenty into test arenas consisting of cylindrical plastic containers (15.2 × 10.2 cm) containing moistened dental plaster poured to a height of 0.75 cm and inner walls coated with a thin layer of Insect-a-Slip. An overturned vial cap (11 × 4 mm) with a hole cut into the side was provided for harborage. Water was provided for the duration of the study via a vial cap completely filled with cotton and saturated with deionized water. The ants were starved for 24 h prior to the start of the test. 

**Figure 3:**
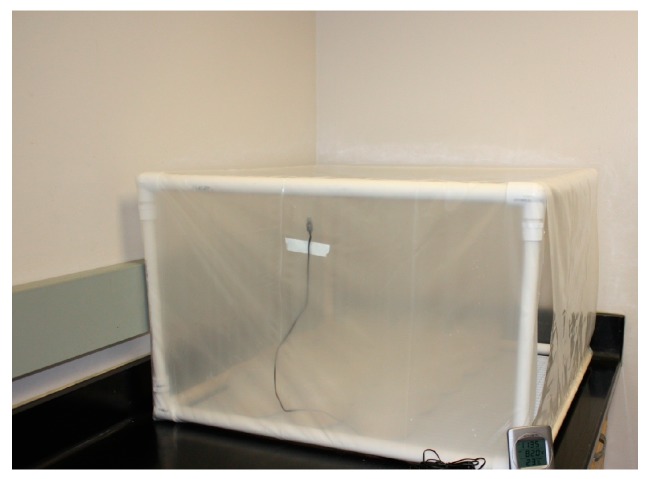
Humidified test chamber for conducting *Nylanderia fulva* bait bioassays. The chamber consists of a polyvinyl chloride (PVC) frame (1.5 × 1.5 × 1.5 m) covered with plastic sheeting. A data logger recorded temperature and humidity within the chamber. The bottom of the chamber contains a water filled pan that is covered by a plastic grid. The individual bioassay arenas are placed atop the grid.

To conduct the bioassays, ~250 mg of bait from an unopened container was measured into a vial cap, and a single cap was placed into each arena. Control arenas received only cotton stuffed vial caps saturated with 10% sucrose solution. Test arenas were placed into a test chamber consisting of a polyvinyl chloride (PVC) frame (1.5 × 1.5 × 1.5 m) covered with plastic sheeting (Figure 3). To maintain adequate humidity, a water-filled tray was placed into the chamber. A plastic grid (1.5 × 1.5 m) was placed over the tray as a surface on which to place the test arenas.

Bioassays were conducted within 2 weeks of *N. fulva* collection in the spring (March–May), summer (June–August), and fall (September–November), presuming that laboratory results would reflect dietary preferences of *N. fulva* in the field. All replications within a season were from a single field collected colony. Each bait test was replicated 10 times for a total of 30 replicates per bait except Amdro^®^ Pro and Niban^®^ Fine Granule which were not tested in the spring and Advion^®^ Ant Gel which was not tested in the fall, as these baits were not available at the time of testing. Bioassays were not conducted during the winter months because *N. fulva* foraging is typically reduced during colder weather. Fifteen commercially available insecticide baits were tested (Table 2). Baits included granular and gel or liquid formulations. All bait treatments were compared to a control of 10% sucrose solution.

To test acceptance of the baits, the numbers of ants on the surface of the bait were recorded at 10, 20, 30, 40, 50, and 60 min after placing baits into the test arenas. Ant counts for each time point were summed to create an acceptance count over all time points. Normality, homoscedasticity, and independence of the data for acceptance counts were evaluated by Shapiro-Wilk, Levene’s, and Durbin-Watson tests, respectively, for all subsequent experiments. Since the data were not normally distributed even after transformation, the acceptance counts were assigned a rank-score of “1” if ant counts were between 0–20, “2” if counts were between 21–40, “3” if counts were between 41–60, “4” if counts were between 61–80, and “5” if counts were between 81–100. Rank-scores among the baits were compared within each season using a one-way analysis of variance (ANOVA) and Tukey’s HSD test (α = 0.05) [13,14]. Results are reported as mean acceptance counts. After bait acceptance was evaluated, the same test arenas, still containing ants, water, and bait were returned to the test chamber to determine bait efficacy and speed of mortality. No additional food was added to the test arenas. The numbers of live ants in each test arena were recorded daily for 7 days or until 100% mortality. If 100% mortality was not achieved in 7 days, the test was terminated and mortality was recorded as 8+ days. 

Delayed toxicity allows optimal bait feeding, recruitment, and transfer of the toxicant to nestmates [15]. Rust et al. [15] found that for *Linepithema humile* Mayr, 1–4 days of exposure to bait caused maximum foraging worker mortality; thus, 3 days after treatment (DAT) and days to 100% mortality were selected as the time intervals for data analyses. Percent mortality at 3 DAT and number of days until 100% mortality data were not normally distributed and were not normalized after transformation. The percentage of dead ants at 3 DAT was assigned a rank-score of “1” if percent mortality was between 0–20, “2” if percent mortality was between 21–40, “3” if percent mortality was between 41–60, “4” if percent mortality was between 61–80, and “5” if percent mortality was between 81–100. Rank-scores for number of days until 100% mortality were assigned the following: “5” = 0 to 1 days to 100% mortality, “4” = 2 to 3 days, “3” = 4 to 5 days, “2” = 6 to 7 days, and “1” = 8+ days to 100% mortality [14]. The rank-scores for percent mortality at 3 DAT and the number of days until 100% mortality were each analyzed using a one-way ANOVA for each season with bait as the main factor [13]. If significant, means were separated using Tukey’s HSD test (α = 0.05). Results are reported as mean rank-scores.

## 3. Results

### 3.1. Contact Insecticide Repellency

**Figure 4:**
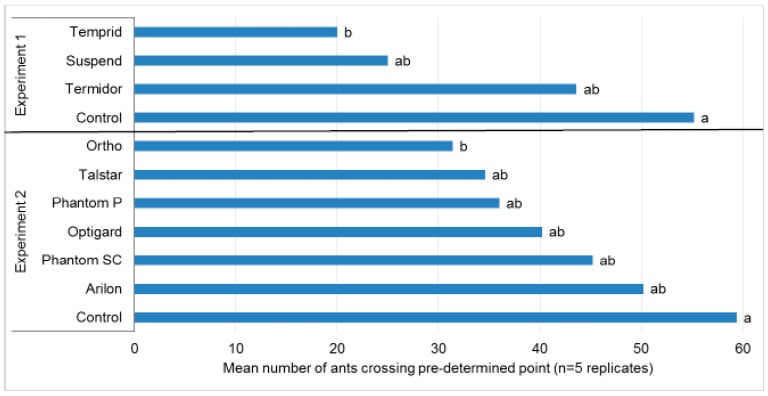
Mean number of *Nylanderia fulva* crossing a predetermined point on a painted wood panel treated with insecticide or water (control) (n = 5 replicates). Counts were taken for 1 min every 30 min for 2 h. Means in the same experiment followed by the same letter are not significantly different (Tukey’s HSD test, α = 0.05) (Experiment 1: *F* = 3.55, df = 3, 16, *P* = 0.0385, Experiment 2: *F* = 2.69, df = 6, 28, *P* = 0.0342).

There were significant differences in foraging counts among treatments for Experiment 1 (*F* = 3.55, df = 3, 16, *P* = 0.0385) and Experiment 2 (*F* = 2.69, df = 6, 28, *P* = 0.0342). No product completely repelled *N. fulva,* as some ants in each treatment traversed the treated surface to obtain food and water (Figure 4). However, over the initial 2 h of Experiment 1, significantly fewer ants crossed surfaces treated with Temprid^®^ compared to the control, but the numbers of ants crossing Temprid^®^ were not significantly different from Termidor^®^ or Suspend^®^. In Experiment 2, only Ortho^®^ was repellent (Figure 4).

### 3.2. Contact Insecticide Efficacy

**Figure 5:**
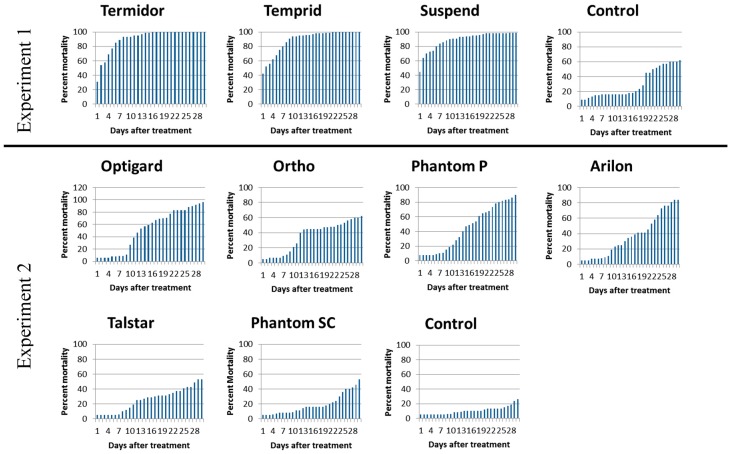
Daily percent mortality of *Nylanderia fulva* workers over 30 days in a bioassay requiring ants to cross a painted wood panel treated with insecticide or water (control) (n = 5 replicates).

There were significant differences in percent mortality among treatments for Experiment 1 (*F* = 34.8, df = 3, 15, *P* < 0.0001). Suspend^®^, Termidor^®^, and Temprid^®^ provided 90–94% worker mortality 10 DAT compared to 16.3% mortality in the control (Table 3, Figure 5). Treatments with Termidor^®^ and Temprid^®^ resulted in 100% mean mortality in 13.4 and 19.0 days, respectively.

In the second experiment, the ANOVA for main factor treatment was not significant at 10 DAT, (*F* = 0.83, df = 6, 28, *P =* 0.5545), but was significant at 28 DAT (*F* = 3.20, df = 6, 28, *P =* 0.0161). Optigard^®^ had significantly higher mortality compared to the control; however, all of the insecticides were not significantly different from each other (Table 3).

An unexpected observation was that in the Termidor^®^ and Temprid^®^ treatments queens lived ~2 days after all workers had died. Thus, any transference of active ingredients associated with these contact insecticide applications appears to have minimal impact on queens, which may help explain why “spraying only” is the least effective method of control for *N. fulva*. In the Suspend^®^ treatment, mortality did not reach 100% by the end of the study and the queens were able to survive for at least 30 days with ~20 workers to tend them. Workers were observed foraging across the treated surface for the entire 30 days.

### 3.3. Bait Acceptance

The ANOVA for each season tested was significant and summarized in Table 4. Competing food sources can reduce bait acceptance, and in this study the sucrose control proved to be more acceptable than the majority of the baits tested. When compared to sucrose, ant acceptance of Advion^®^ Ant Gel in the spring was not significantly different. Also in the spring, most of the granular baits were less accepted than liquid or gel. In summer, only InTice^™^ gel and Advion^®^ gel was significantly more accepted compared to the sucrose control and all other baits. During fall, only InTice^™^ gel was significantly more accepted than the sucrose control and all other baits. Advion^®^ Ant Gel was not tested during the fall (Table 4). During the spring and fall, Amdro^®^ Pro, Esteem^®^, and Extinguish^®^, formulated with oil, were among the least accepted baits by *N. fulva*; however, during the summer, *N. fulva* seemed to accept a wider range of bait formulations relative to the control (Table 4).

### 3.4. Bait Efficacy

The ANOVA for each season tested was significant and summarized in Table 5 and Table 6. In the spring, Maxforce^®^ Fine Granular, Maxforce^®^ Ant Killer Bait Gel, Maxforce^®^ granular, Extinguish^®^ Pro, Advion^®^ Maxforce^®^ Complete, Advance^®^ Carpenter Ant, InTice™, and Esteem^®^ had significantly greater mortality at 3 DAT compared to the control and other baits. Of these baits, only Advion^®^ and Maxforce^®^ Fine Granular had ant acceptance scores that were not significantly different than the sucrose control (Table 4).

In summer, all of the baits had mortality scores that were significantly different than the control except 381B Advance, a liquid bait. Exposure to Maxforce^®^ Complete and Amdro^®^ Pro resulted in the highest mortality 3 DAT (Table 5), and had acceptance scores that were not significantly different than the control (Table 4).

In the fall, Maxforce^®^ Ant Killer Bait Gel, Maxforce^®^ Complete, Amdro^®^ Pro, Maxforce^®^ Fine Granular, Niban^®^ FG, Advance^®^ Carpenter Ant Bait, and Optigard^®^ resulted in the highest mortality at 3 DAT (Table 5) compared with the control. However, all of these baits, except Niban^®^ FG, had significantly lower acceptance scores than the control (Table 4).

Amdro^®^ Pro, Maxforce^®^ Complete, Maxforce^®^ Fine Granular, and Maxforce^®^ Granular all contain the active ingredient hydramethylnon. Maxforce^®^ Ant Killer Bait Gel and Advance^®^ 375A in the spring and Maxforce^®^ Complete in the summer and fall had the fewest days to 100% mortality (Table 6). Ant baits with the active ingredient boric acid (381B Advance^®^ and InTice™ Smart Ant Gel) took greater than 6 days to achieve 100% mortality.

**Figure 6:**
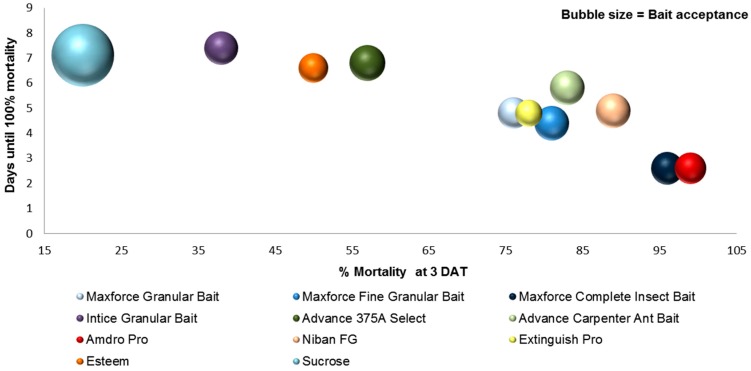
Laboratory assay results for bait performance criteria of acceptance, days until 100% mortality, and percent mortality at 3 DAT for commercially available granular ant baits combined into a single graph to allow direct comparisons. Baits indicated by bubbles in lower, right quadrant and with largest diameter are those with highest mortality at 3 DAT, fewest days to 100% mortality, and highest acceptance. Sucrose control is blue, top left corner.

**Figure 7:**
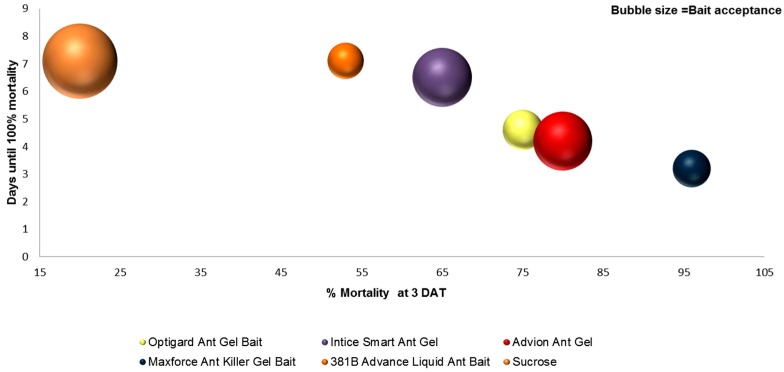
Laboratory assay results for bait performance criteria of acceptance, days until 100% mortality, and percent mortality at 3 DAT for commercially available liquid or gel ant baits combined into a single graph to allow direct comparisons. Baits indicated by bubbles in lower, right quadrant and with largest diameter are those with highest mortality at 3 DAT, fewest days to 100% mortality, and highest acceptance. Sucrose control is burnt orange, top left corner.

When selecting a product for control of *N. fulva*, multiple performance criteria must be considered. For example, although InTice™ Smart Ant Gel had the highest acceptance scores in the summer and fall, it was not significantly different from the control in the number of days until 100% mortality (Table 6). Assuming rapid suppression of the foraging ant population is the objective, then the ideal bait would be highly acceptable and fast acting. By combining the efficacy data, percent mortality at 3 DAT (x-axis), and number of days until 100% mortality (y-axis) with the acceptance data (bubble size = acceptance score), we arrive at a three-dimensional representation that shows that the best performing products using these performance criteria were Amdro^®^ Pro, Maxforce^®^ Complete Insect Bait, and Maxforce^®^ Ant Killer Bait Gel (Figure 6 and Figure 7).

## 4. Discussion

In this study, we examined the repellency and efficacy of commonly used insecticide products in Florida under experimental conditions that are similar to the way in which *N. fulva* would interact with the treated exterior perimeter of a structure. It was not unexpected that products such as Termidor^®^, Phantom^®^, and Phantom^®^ aerosol would not repel *N. fulva* from traversing a treated surface to forage, as these products contain known non-repellent active ingredients [15,16,17,18]. However, in this closed-system bioassay, none of the products tested were truly repellent and only Temprid^®^ and Ortho^®^ resulted in significantly fewer ants crossing an insecticide treated surface to receive food and water compared to the untreated control.

It was expected that foraging workers would be exposed to the insecticide and die, requiring additional workers to forage. Thus, eventually all workers would succumb to the effects of the insecticide. We expected that without workers to tend the queens, they would eventually die of starvation [18]. Alternatively, with non-repellent, slow-acting insecticides such as Termidor^®^ and Phantom^®^, the foraging ants would contact the insecticide and transfer the toxicant to non-foraging nestmates resulting in worker and queen mortality [18,19].

Most of the insecticides tested did not provide 100% mortality by the end of the 30-day study, even when worker ants were forced to cross the treated surface for food and water, and queens survived for ~2 days after all the workers died in the Termidor^®^ and Temprid^®^ treatments. The wood panels used in the study, even though painted and treated with the highest concentration of insecticide allowed by the label, may not have had sufficient active ingredient bioavailability. Substrate effects on pesticide efficacy have been demonstrated previously. Wagner and Strawn [20] found that knockdown of *L. humile* was less than 90% one day after treating concrete with chlorpyrifos compared to six months on other substrates. A comparison of substrate effect on the efficacy of Termidor^®^, Phantom^®^, and Talstar^®^ showed that worker mortality of *Monomorium pharaonis* (L.) was less on concrete than hardwood mulch [21].

Structures in Florida are constructed of a variety of building materials and future studies should include additional commonly used construction materials. However, even if efficacy is improved by choice of a suitable substrate, laboratory bioassays do not account for environmental conditions such as overspray from irrigation, intense UV radiation, and high temperatures that may degrade the active ingredient. Furthermore, this bioassay did not account for insect behaviors that may impact the effectiveness of contact insecticides for *N. fulva* control. In this study, at least some foraging ants contacted the insecticide treated surface daily to obtain food and water. Under natural conditions, *N. fulva* would likely have access to alternative food resources and could avoid exposure to the insecticide on the exterior perimeter of a structure. In addition, the *N. fulva* queens in this study survived without workers for ~2 days. As a polydomous tramp ant species lacking intraspecific aggression, an *N. fulva* queen without workers in the field may relocate. In the laboratory, queens from one colony have been placed successfully with workers from another colony, indicating the possibility of colonies accepting new queens in field situations [22]. The results of this study suggest further work can be conducted on the success of queen adoption by new nests. Additional studies may also investigate mechanisms that may confer reduced susceptibility of *N. fulva* to insecticides on treated surfaces.

In our bait studies, the control of 10% sucrose solution proved to be more accepted than almost all other baits except Advion^®^ Ant Gel and InTice^™^ Smart Ant Gel. Although InTice^™^ Smart Ant Gel had the highest acceptance score, it did not induce significant mortality. This is not surprising, as the active ingredient, borax (=sodium tetraborate decahydrate), is known to be slow acting [23]. It was surprising that 381 B Advance^®^, a liquid formulation, was not highly accepted. The active ingredient 1.3% borax is also the active ingredient in InTice™ Smart Ant Gel (5% borax), a bait that was highly accepted. While the inert ingredients are proprietary and unknown, the advertising for InTice™ Smart Ant Gel claims that it is “super sweet,” suggesting it may have a higher concentration of sugar, and therefore, induce more feeding. In general, gel baits were more accepted by *N. fulva* in this study. However, their utility in an IPM program is limited. The volume of gel bait required to impact a *N. fulva* infestation would be costly and aesthetically unappealing. The recent label amendment to Maxforce^®^ Quantum (imidacloprid, Bayer Environmental Science, Research Triangle Park, NC, USA) allows the gel bait to be mixed into a 25% sucrose solution without compromising efficacy in an effort to satisfy the need for large quantities of bait.

In our study, most granular baits were less accepted than the liquid sucrose control by *N. fulva*. However, of the granular baits, Advance^®^ 375A (spring, fall) and Advance^®^ Carpenter Ant Bait (summer) had some of the highest acceptance scores. These baits contain both protein and carbohydrate constituents. Stanley [24] also recommended protein-based baits for some crazy ant species. Advance^®^ 375A and Advance^®^ Carpenter Ant Bait both contain the active ingredient abamectin, while baits that generally resulted in the highest mortality 3 DAT contained hydramethylnon. Granular baits containing the active ingredient boric acid were the poorest performing baits based on the criteria defined in this study of high percent mortality by 3 DAT and 100% mortality in 7 days. However, boric acid baits did provide approximately 50% mortality by 3 DAT and if the study had been conducted for a longer period of time, 100% mortality may have eventually occurred. Therefore, boric acid-containing baits may have utility in IPM programs in sensitive environments where the use of other classes of chemical insecticides is limited.

The oil-containing baits, developed to be attractive to red imported fire ants, *S. invicta*, show variable acceptance by *N. fulva*. During the spring and fall, oil-based baits were less accepted, while during the summer, acceptance was not different than sucrose controls. Stanley and Robinson [25] showed that the black crazy ant, *Paratrechina longicornis*, was not attracted to oil containing baits. However, Zenner-Polania [2] used a mixture of pork lard, corn bran, fish meal, sugar, proprionic acid, and carbaryl as a *N. fulva* bait that “gave good ant control for at least two months.”

Amdro^®^ Pro, an oil-based bait, was not highly accepted by *N. fulva*, yet resulted in a high percent mortality at 3 days (Table 5). In the small arena, no-choice assay, ants were unable to avoid the bait. A similar phenomenon was reported by Oi [26] who documented significant *N. fulva* brood reduction after exposure to the insect growth regulators pyriproxyfen and (S)-methoprene, but also noted repellency to the active ingredients. Under field conditions where ants have dietary choices, a bait formulated with an unacceptable matrix or repellent active ingredient will not provide an acceptable level of efficacy.

## 5. Conclusions

Insecticides are an integral part of an IPM program. Temprid^®^, Termidor^®^, and Suspend^®^ were the only commercially available spray insecticides that provided acceptable efficacy in laboratory studies. Considering bait acceptance, delayed mortality, and efficacy together allows the direct comparison of commercially available products included in this study. These data suggest that Maxforce^®^ Ant Killer Bait Gel and the granular baits Amdro^®^ Pro and Maxforce^®^ Complete may be effective bait products for the suppression of *N. fulva*; however, acceptance is still less than the sucrose-solution controls. Further field testing is suggested. These data also suggest that the active ingredients hydramethylnon and fipronil could be very effective against *N. fulva*, especially when combined with a matrix optimized for attractiveness and palatability.

## Figures and Tables

**Table 1:** Nine commercially available contact insecticides tested for repellency and efficacy against *Nylanderia fulva* in laboratory bioassays.

Trade Name	Active Ingredient	Concentration of Active Ingredient in Dilution	Marketed as Repellent or Non-Repellent	Manufacturer
Arilon^®^	Indoxacarb	0.10%	Non-repellent	Syngenta, Greensboro, NC, USA
Optigard^®^	Thiamethoxam	0.10%	Non-repellent	Syngenta, Greensboro, NC, USA
Ortho^®^	Acephate	0.07%	Repellent	Monsanto, San Ramon, CA, USA
Phantom^®^ aerosol	Chlorfenapyr	0.50%	Non-repellent	BASF, Research Triangle Park, NC, USA
Phantom^®^ SC	Chlorfenapyr	0.50%	Non-repellent	BASF, Research Triangle Park, NC, USA
Suspend^®^	Deltamethrin	0.03%	Repellent	Bayer, Research Triangle Park, NC, USA
Talstar^®^	Bifenthrin	0.062%	Repellent	FMC, Philadelphia, PA, USA
Temprid^®^	Imidacloprid + Beta cyfluthrin	0.10% + 0.05%	Non-repellent and repellent active ingredients	Bayer, Research Triangle Park, NC, USA
Termidor^®^	Fipronil	0.06%	Non-repellent	BASF, Research Triangle Park, NC, USA

**Table 2:** Insecticide baits tested for acceptance and efficacy against *Nylanderia fulva* in laboratory bioassays.

Product	Active Ingredient	Formulation Type	Manufacturer
InTice™ Granular Ant Bait	Orthoboric acid	Granular	Rockwell Labs, Ltd., North Kansas City, MO, USA
381B Advance^®^ Liquid Bait	Borax	Liquid	BASF, Research Triangle Park, NC, USA
Advance^®^ 375A Select	Abamectin	Granular	BASF, Research Triangle Park, NC, USA
Advance^®^ Carpenter Ant Bait	Abamectin	Granular	BASF, Research Triangle Park, NC, USA
Advion^®^ Ant Gel	Indoxacarb	Gel	Dupont, Wilmington, DE, USA
Amdro^®^ Pro	Hydramethylnon	Granular	BASF, Research Triangle Park, NC, USA
Esteem^®^ Ant Bait	Pyriproxyfen	Granular	Valent USA Corporation, Walnut Creek, CA, USA
Extinguish^®^ Professional	Methoprene	Granular	Wellmark International, Schaumberg, IL, USA
InTice™ Smart Ant Gel	Borax	Gel	Rockwell Labs, Ltd., North Kansas City, MO, USA
Maxforce^®^ Ant Killer Bait Gel	Fipronil	Gel	Bayer Environmental Science, Research Triangle Park, NC, USA
Maxforce^®^ Complete Bait	Hydramethylnon	Granular	Bayer Environmental Science, Research Triangle Park, NC, USA
Maxforce^®^ Fine Granular Bait	Hydramethylnon	Granular	Bayer Environmental Science, Research Triangle Park, NC, USA
Maxforce^®^ Granular Bait	Hydramethylnon	Granular	Bayer Environmental Science, Research Triangle Park, NC, USA
Niban^®^ Fine Granular Bait	Orthoboric acid	Granular	Nisus Corporation, Rockford, TN, USA
Optigard^®^ Ant Gel Bait	Thiamethoxam	Gel	Syngenta Crop Protection, Greensboro, NC, USA

**Table 3:** Mean percent *Nylanderia fulva* mortality at 10 and 17 days after treatment (DAT) (Experiment 1) or 28 DAT (Experiment 2) when control mortality approached 20% in a bioassay requiring ants to cross a painted wood panel treated with insecticide or water (control). Insecticides were tested in two groups (Experiment 1 and Experiment 2) (n = 5 replicates for each insecticide, except for n = 4 replicates in Experiment 1).

Percent Mortality						
**Experiment**	**10 DAT**			**17 DAT**		
Experiment 1	Temprid	94.0	a ^1^	Termidor	100.0	a
	Termidor	92.8	a	Temprid	97.6	a
	Suspend	90.0	a	Suspend	94.8	a
	Control	16.3	b	Control	20.0	b
	**10 DAT**			**28 DAT**		
Experiment 2	Optigard	27.0		Optigard	92.0	a
	Ortho	21.0		Phantom P	84.0	ab
	Phantom P	19.0		Arilon	80.6	ab
	Arilon	19.0		Ortho	60.0	ab
	Talstar	15.0		Talstar	49.0	ab
	Phantom SC	9.0		Phantom SC	42.0	ab
	Control	6.0		Control	19.0	b

^1^ Insecticides in the same experiment followed by the same letter are not significantly different (Tukey’s HSD α = 0.05). Experiment 1 10 DAT *F* = 34.8, df = 3, 15, *P* < 0.0001; 17 DAT: *F* = Infinity, df = 3, 15, *P* = 0.0001. Experiment 2 10 DAT: *F* = 0.83, df = 6, 28 *P* = 0.5545; 28 DAT: *F* = 3.20, df = 6, 28, *P* = 0.0161. Analyses were conducted on rank-scores.

**Table 4:** *Nylanderia fulva* acceptance of 15 commercially available baits in no-choice laboratory assays over three seasons (2009–2010) (n = 10 replications for each bait per season).

Mean Bait Acceptance Counts								
Bait	Spring		Bait	Summer		Bait	Fall	
Advion (g) ^1^	19.9	a ^2^	InTice (g)	7.7	a	InTice (g)	24.8	a
Control (l)	9.1	abc	Advion (g)	5.7	ab	InTice (gr)	4.1	abc
InTice (g)	7.7	bc	Advance Carpenter Ant (gr)	3.9	bc	Control (l)	3.9	b
Advance 375A Select (gr)	4.1	cd	Optigard (g)	2.8	cd	Advance 375A Select (gr)	2.7	bcd
Maxforce Fine Granular (gr)	3.3	cd	Maxforce Complete (gr)	2.2	cd	Niban FG (gr)	1.9	bcd
Maxforce Ant Killer (g)	2.5	de	InTice (gr)	1.9	cd	Maxforce Fine Granular (gr)	2.8	bd
Optigard (g)	1.4	de	Control (l)	1.8	cd	Advance Carpenter Ant (gr)	2.3	cd
Maxforce Granular Bait (gr)	0.5	de	Niban FG (gr)	1.3	cd	Optigard (g)	2.2	cde
381B Advance (l)	0.4	de	Esteem (gr)	0.7	cd	Maxforce Granular Bait (gr)	1.2	cde
Advance Carpenter Ant (gr)	0.4	de	Amdro Pro (gr)	0.5	cd	Maxforce Complete (gr)	1.1	cde
InTice (gr)	0.1	e	Extinguish Pro (gr)	0.4	cd	381B Advance (l)	1.0	cde
Maxforce Complete (gr)	0.0	e	Maxforce Fine Granular (gr)	0.3	cd	Amdro Pro (gr)	0.6	cde
Extinguish Pro (gr)	0.0	e	Advance 375A Select (gr)	1.1	d	Maxforce Ant Killer (g)	0.5	de
Esteem (gr)	0.0	e	Maxforce Ant Killer (g)	0.9	d	Esteem (gr)	0.5	de
Niban FG (gr)	NT		381B Advance (l)	0.3	d	Extinguish Pro (gr)	0.0	e
Amdro Pro (gr)	NT ^3^		Maxforce Granular Bait (gr)	0.3	e	Advion (g)	NT	
	*F* = 16.82			*F* = 11.46			*F* = 26.50	
	df = 13, 126			df = 15, 144			df = 14, 134	
	*P* < 0.0001			*P* < 0.0001			*P* < 0.0001	

^1^ Key: g = gel, l = liquid, gr = granular. ^2^ Mean acceptance counts in the same season followed by the same letter are not significantly different (Tukey’s HSD test, α = 0.5). Analyses were conducted on rank-scores. ^3^ Not tested.

**Table 5:** Mean percent mortality rank-score of *Nylanderia fulva* at 3 days after continuous exposure to 15 commercially available baits in no-choice laboratory assays over three seasons (2009–2010) (n = 10 replications for each bait per season).

Mean Percent Mortality at 3 DAT Rank-Score ^1^								
**Bait ^2^**	**Spring**		**Bait**	**Summer**		**Bait**	**Fall**	
Maxforce Fine Granular (g)	4.9	a ^3^	Maxforce Complete (gr)	5.0	a	Maxforce Ant Killer (g)	5.0	a
Maxforce Ant Killer (g)	4.9	a	Amdro Pro (gr)	5.0	a	Maxforce Complete (gr)	5.0	a
Maxforce Granular (gr)	4.9	a	Niban FG (gr)	4.8	ab	Amdro Pro (gr)	5.0	a
Extinguish Pro (gr)	4.8	a	Maxforce Ant Killer (g)	4.7	abc	Maxforce Fine Granular (g)	4.9	a
Advion (g)	4.6	a	Maxforce Granular (gr)	4.7	abc	Niban FG (gr)	4.7	ab
Maxforce Complete (gr)	4.5	ab	Optigard (g)	4.6	abc	Advance Carpenter Ant (gr)	4.3	abc
Advance Carpenter Ant (gr)	4.4	ab	Advance Carpenter Ant (gr)	4.6	abc	Optigard (g)	4.2	abcd
InTice (g)	4.0	abc	Advance 375A Select (gr)	4.5	abcd	Extinguish Pro (gr)	4.1	abcde
Esteem (gr)	3.8	abc	InTice (g)	3.9	abcd	381B Advance (l)	3.9	abcde
Optigard (g)	3.3	de	Advion (g)	3.7	abcde	InTice (g)	3.1	bcdef
Advance 375A Select (gr)	1.4	d	Extinguish Pro (gr)	3.5	abcde	Maxforce Granular(gr)	2.7	cdef
381B Advance (l)	1.2	d	Maxforce Fine Granular (g)	3.2	bcde	Advance 375A Select (gr)	2.6	cdef
Control (l)	1.1	d	InTice (gr)	3.1	cde	InTice (gr)	2.5	def
InTice (gr)	0.1	e	Esteem (gr)	2.9	de	Control (l)	2.4	ef
Niban FG (gr)	NT ^4^		381B Advance (l)	2.2	ef	Esteem (gr)	1.8	F
Amdro Pro (gr)	NT		Control (l)	1.0	f	Advion (g)	NT	
	*F* = 33.31			*F* = 11.39			*F* = 10.20	
	df = 13, 126			df = 15, 144			df = 14, 135	
	*P* < 0.0001			*P* < 0.0001			*P* < 0.0001	

^1^ Percent mortality rank-scores: “1” = 0–20%, “2” = 21–40%, “3” = 41–60%, “4” = 61–80%, and “5” = 81–100%. ^2^ Key: g = gel, l = liquid, gr = granular. ^3^ Mean rank-scores in the same season followed by the same letter are not significantly different (Tukey’s HSD test, α = 0.5). ^4^ Not tested.

**Table 6:** Mean ranked days until 100% mortality of *Nylanderia fulva* after continuous exposure to 15 commercially available baits in no-choice laboratory assays over three seasons (2009–2010) (n = 10 replications for each bait per season).

Mean Number of Days Until 100% Mortality Rank-Score ^1^								
**Bait ^2^**	**Spring**		**Bait**	**Summer**		**Bait**	**Fall**	
Maxforce Ant Killer (g)	4.2	a ^3^	Maxforce Complete (gr)	4.0	a	Maxforce Complete (gr)	4.2	a
Advance 375A Select (gr)	4.1	a	Amdro Pro (gr)	3.8	ab	Maxforce Ant Killer (g)	4.0	ab
Extinguish Pro (gr)	3.6	ab	Optigard (g)	3.6	ab	Amdro Pro (gr)	4.0	ab
Maxforce (gr)	3.5	ab	Advion (g)	3.3	abc	Maxforce Fine Granular (g)	3.3	ab
Maxforce Complete (gr)	3.3	abc	Maxforce Ant Killer (g)	3.0	abcd	Niban FG (gr)	3.0	bcd
Advion (g)	3.0	bc	Maxforce Fine Granular (g)	2.9	abcd	Extinguish Pro (gr)	2.6	cde
Optigard (g)	3.0	bc	Extinguish Pro (gr)	2.6	abcde	Advance Carpenter Ant (gr)	2.5	cdef
Maxforce Fine Granular (g)	2.9	bc	Niban FG (gr)	2.6	abcde	Maxforce (gr)	2.2	def
InTice (g)	2.6	bc	Advance 375A Select (gr)	2.4	bcdef	Optigard (g)	2.1	def
Advance Carpenter Ant (gr)	2.6	bc	Advance Carpenter Ant (gr)	1.9	cdef	381B Advance (l)	2.1	def
Esteem (gr)	2.3	cd	Esteem (gr)	1.7	def	InTice (g)	2.0	defg
Control (l)	1.0	d	InTice (g)	1.4	ef	InTice (gr)	1.8	efg
381B Advance (l)	1.0	d	InTice (gr)	1.2	ef	Esteem (gr)	1.5	fg
InTice (gr)	1.0	d	Control (l)	1.0	f	Control (l)	1.0	g
Niban FG (gr)	NT ^4^		Maxforce (gr)	0.3	e	Advance 375A Select (gr)	1.0	g
Amdro Pro (gr)	NT		381B Advance (l)	0.3	e	Advion (g)	NT	
	*F* = 18.37			*F* = 10.75			*F* = 22.47	
	df = 12, 112			df = 14, 135			df = 14, 135	
	*P* < 0.0001			*P* < 0.0001			*P* < 0.0001	

^1^ Number of days until 100% mortality rank-scores: “5” = 0–1, “4” = 2–3, “3” = 4–5, “2” = 6–7, and “1” > 8. ^2^ Key: g = gel, l = liquid, gr = granular. ^3^ Mean rank-scores in the same season followed by the same letter are not significantly different (Tukey’s HSD test, α = 0.5). ^4^ Not tested.
